# Compulsory Licenses for Cancer Drugs: Does Circumventing Patent Rights Improve Access to Oncology Medications?

**DOI:** 10.1200/JGO.2016.005363

**Published:** 2016-06-29

**Authors:** Cinthia Leite Frizzera Borges Bognar, Brittany L. Bychkovsky, Gilberto de Lima Lopes

**Affiliations:** **Cinthia Leite Frizzera Borges Bognar**, Centro Paulista de Oncologia, and Oncoclinicas do Brasil; **Gilberto de Lima Lopes Jr**, Oncoclinicas Group, Sao Paulo, Brazil; **Brittany L. Bychkovsky**, Dana-Farber Cancer Center Harvard Medical School, Boston, MA; and **Gilberto de Lima Lopes Jr**, Johns Hopkins University School of Medicine, Baltimore, MD.

## Abstract

Worldwide, there are enormous inequities in cancer control that cause poor outcomes among patients with cancer who live in low- and middle-income countries (LMICs). One of the biggest challenges that oncology faces today is how to increase patient access to expensive, but life-saving, therapies in LMICs. Access to cancer medications in LMICs is a major problem, especially in recent years, as the costs of these therapies continue to rise exponentially. One mechanism available to LMICs to improve access to cancer medications allows a country to pursue a compulsory license for a given drug. Here, we will review how the legal framework in the World Trade Organization's Trade-Related Aspects of Intellectual Property Rights Agreement and the Doha Declaration supports countries to circumvent patent laws and acquire compulsory licenses for essential medicines. We will also discuss the current and future role of compulsory licenses in oncology and how compulsory licenses may improve access to cancer drugs in LMICs.

## INTRODUCTION

Cancer is one of the leading global causes of morbidity and mortality, with approximately 14 million new cases and 8.2 million deaths in 2012.^1^ Worldwide, there are enormous inequities in cancer control that result in poor outcomes for patients in low- and middle-income countries (LMICs).^2,3^ More than 60% of new cases occur in Africa, Asia, and Latin America, and these regions account for 70% of the world’s cancer deaths.^4^ The burden of cancer in LMICs also significantly impacts the economy of these regions, yet only 6% of global resources for cancer are spent in the developing world.^5,6^ In the near future, this gap in cancer care between LMICs and high-income regions is predicted to grow. By 2020, cancer is likely to kill more than twice as many people worldwide as in 2000 and the death rate in LMICs will be at least five times greater than in high-income countries.^7^

Cancer outcomes are clearly related to where one lives.^8^ Patients with breast cancer in the United States have a 5-year overall survival of 84%, whereas, in Gambia, 5-year overall survival is only 12%.^7^ One of the biggest challenges that faces the world of oncology is how LMICs will address the rising burden of cancer in their regions—this includes difficult decisions of when to offer expensive cancer therapies to patients and how to best organize cancer prevention programs within health systems that have suboptimal infrastructure and support.^2,3,5^ Access to cancer drugs for LMICs is a growing problem because many new medications in oncology are exorbitantly expensive and prices have risen in recent years: the average price of a cancer therapy has doubled from US$5,000 per month in 2003 to US$10,000 per month in 2013 and continues to rise.^9^ High costs are a major, and often insurmountable, barrier in poor countries for which the price of standard cancer medications are simply too high relative to the national and individual income.^10^

Countries design and enforce patent laws to protect intellectual property of pharmaceutical and other products and services. Many authors argue that this is an important way to incentivize drug development, as it creates a de facto monopoly, which, in theory, allows medications to be priced in such a way that covers the costs of production, recoups investment in research and development, and, ideally, brings in profits to innovative companies and adequate financial returns for their investors. New medications covered under patent laws are priced for high-income countries, which almost always makes them unaffordable to large numbers of patients and health systems in LMICs.

## WTO TRADE-RELATED ASPECTS OF INTELLECTUAL PROPERTY RIGHTS AGREEMENT AND THE DOHA DECLARATION

Before 1995, there was significant variability across countries in how patented medications were regulated. This changed when the World Trade Organization (WTO) and member countries approved the Trade-Related Aspects of Intellectual Property Rights (TRIPS) Agreement, which greatly influenced interpretation of patent laws.^11^ Before TRIPS, more than 40 countries did not offer patent protection for pharmaceutical products and many developing countries that did only offered protection for 5 to 7 years.^11^ The agreement, which went into effect in 1995, required all WTO countries to provide patent protection for a minimum of 20 years, and this included patents for both pharmaceutical processes and products; however, the TRIPS agreement contains provisions that allow individual countries to balance their intellectual property and patents with their own health and development needs.^11^ For example, countries can issue compulsory licenses to make generic medications on the grounds of public interest. This means that a country can produce generic drugs without the consent of the patent holder even when intellectual property rights are still in effect.

After the TRIPS Agreement, the Doha Declaration was adopted in 2001 by the WTO and supported the notion that member states could circumvent patent rights by issuing compulsory licenses that would allow them to access essential medications if these medications were urgently needed to protect the public’s health. The Doha Declaration also contained provisions that allowed LMICs that were without drug manufacturing capabilities to import medications produced elsewhere under compulsory licensing. The pharmaceutical company that owns the original patent still holds the right to its invention and is entitled to compensation under TRIPS, which, therefore, means that governments must first negotiate with the pharmaceutical industry directly to ask to purchase the desired medication at a reduced price or they must request a voluntary license from the pharmaceutical company to manufacture the drug before they can issue a compulsory one.^5,12,13^ Only in situations of an emergency or extreme urgency, such as an epidemic, can governments forgo the process of negotiating with the patent holder.^14^ It was after the Doha Declaration that several countries used compulsory licensing to increase access to HIV/AIDs drugs.^15^

An amendment to the WTO TRIPS agreement allows for least developed countries (LDCs) that are members of the WTO to import generic medications, ignoring both local and international patent laws.^16^ This pharmaceutical product waiver was recently extended until 2033 and applies to 34 WTO member countries.^16,17^ These LDCs can also produce their own generics, but, to date, most countries involved do not have a pharmaceutical generic manufacturing industry. Among all WTO LDCs, only Uganda, Nepal, and Bangladesh have a nascent pharmaceutical industry, and these three countries indeed led the most recent negotiations to get the amendment approved.^18^

## USE OF COMPULSORY LICENSES FOR HIV THERAPY

To date, compulsory licenses have been widely used to enhance access to medications to treat communicable disease, such as HIV, tuberculosis, and malaria, and helped to bring life-saving drugs to patients around the world.^15^ When an Indian manufacturer (Cipla) began to offer HIV/AIDs triple therapy for US$350 per patient per year in 2001, it made international headlines because the patented equivalent had cost US$10,000 to US$15,000 per patient.^12^ As soon as the generic from Cipla became available, governments began to issue compulsory licenses for this medication so that they could purchase the generic.^19^ In May 2007, after negotiations failed with the patent holder, the Brazilian government granted its first compulsory license for the public noncommercial use of efavirenz, an essential HIV antiretrovial.^20^ By using a generic version of efavirenz, the Brazilian government saves approximately US$30 million per year—money that can now be used for other public health needs.^19^

## USE OF COMPULSORY LICENSES FOR HEPATITIS C

In 2013 after the US Food and Drug Administration approved sofosbuvir for hepatitis C, Gilead Sciences, the manufacturer of the drug (note that as of this writing, Gilead is involved in an ongoing dispute with Merck for the patent of sofosbuvir),^21^ was immediately criticized for its high cost as its price is so elevated relative to production cost.^14^ For example, a typical 12-week course of sofosbuvir is US$84,000 per patient, although total production costs are only US$68 to US$136.^22,23^ To circumvent the high cost, efforts in many LMICs were quickly taken to gain access to this medication for patients with hepatitis C virus (HCV). India was one of the first countries to start production of a generic version of sofosbuvir, and a 12-week course is now on the market for US$567.^24,25^

In response to the generic version, Gilead Sciences announced in September 2014 that it granted a voluntary license for sofosbuvir and ledipasvir (a sister drug to sofosbuvir) to 11 Indian drug manufacturers.^17^ The agreement allows these Indian companies to produce and sell generic versions of sofosbuvir and ledipasvir to 91 LMICs^25^; however, the agreement excluded many LMICs, countries in which 73 million people with HCV live.^26^ The licensing agreement left out 46% of people who need HCV treatment worldwide, including those who live in Brazil (2.6 million with HCV), Thailand (1.5 million with HCV), and Morocco (625,000 with HCV).^26^ Countries left out of this agreement need to either negotiate a discounted rate directly with Gilead Sciences or pursue compulsory licenses for sofosbuvir and ledipasvir, both of which take time and delay patient access to these medications.

Brazil, as one of the largest countries in Latin America, does have bargaining power unlike other small LMICs and has now negotiated with Gilead Sciences to purchase sofosbuvir, daclastavir, and simeprevir. Through this agreement, a course of sofosbuvir will cost US$9,425 for 12 weeks of treatment.^27^ After this agreement, Mercosur, a trade collaborative between Argentina, Brazil, Bolivia, Uruguay, Paraguay, and Venezuela, negotiated the purchase of sofosbuvir, daclastavir, and simeprevir with Gilead Sciences in November 2014 at a 90% discount.^27^

The status of sofosbuvir in Egypt is evolving but important to follow, because Egypt has one of the highest rates of hepatitis C infection in the world after nearly 9 million people were infected when a national mass therapy program against schistosomiasis used contaminated needles.^28,29^ The Egyptian government has rejected Gilead Sciences’ application for sofobusvir’s patent, which made it inevitable that generic versions would eventually be sold there. In response to this, Gilead Sciences offered to sell the drug for US$10 a pill or for US$900 for a 12-week course. Subsequent to this, the government dispensed sofosbuvir free to patients, with some restrictions to prevent a black market trade of the bottles. In the first year, 125,000 patients were treated with sofosbuvir. Now, generic versions of sofosbuvir are available for as little as US$4 a pill, and Egypt is introducing these generics into the public health system.^28^

## EXAMPLES OF COMPULSORY LICENSES GRANTED FOR CANCER MEDICATIONS

To date, compulsory licenses have not been widely used in LMICs to increase access to essential medicines for patients with cancer (Table 1); however, there are two important examples in which compulsory licenses were used for cancer drugs in Thailand and India.

**Table 1:**
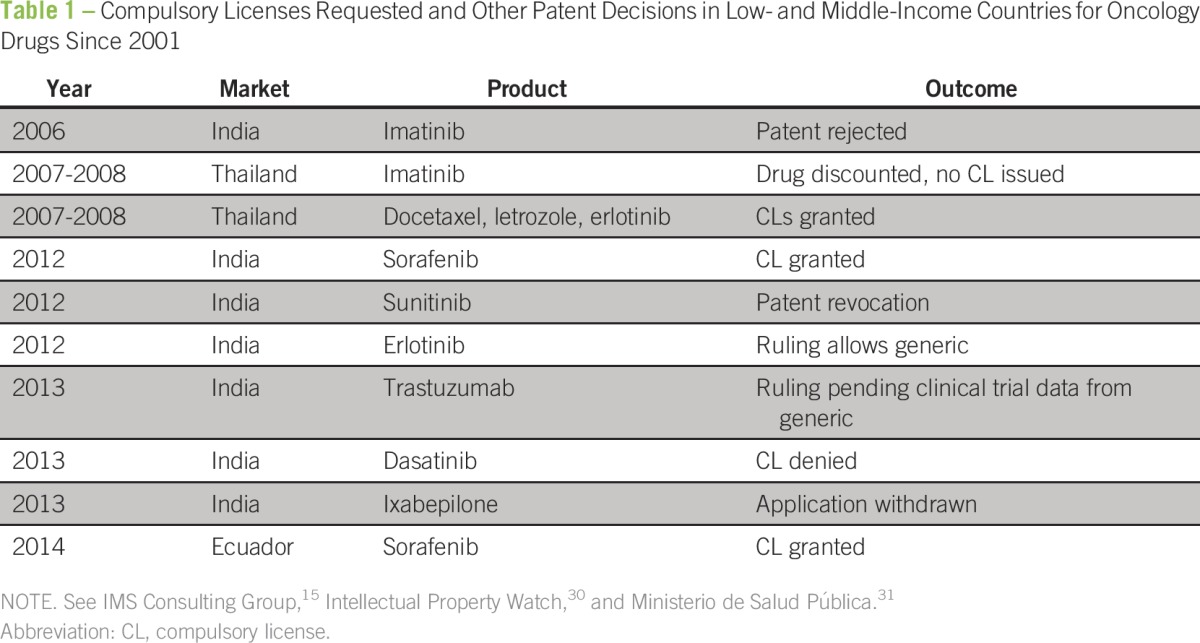
Compulsory Licenses Requested and Other Patent Decisions in Low- and Middle-Income Countries for Oncology Drugs Since 2001

In 2008, the Thai government issued compulsory licenses for erlotinib, letrozole, and docetaxel, and was one of the first countries to grant a compulsory license for a noncommunicable disease.^19^ Introduction of a generic version of letrozole was estimated to save US$88 to US$102 million per year, docetaxel US$46 to US$53 million per year, and erlotinib US$6 to US$8 million per year.^32^ A compulsory license for imatinib was initially pursued, but then canceled after negotiations with Novartis proceeded—Novartis has now made imatinib available to all patients who receive care in the public health system as part of their funded International Patient Assistance Program.^6,33^ The Thai government decision to issue compulsory licenses for oncology drugs coincided with other cost-containment measures and efforts to expand public health coverage.

After compulsory licenses were pursued for erlotinib, letrozole, and doxcetaxel in Thailand, there were clear benefits in terms of reducing drug costs and improving access to treatments for patients with cancer.^6^ For letrozole, the compulsory license reduced the cost per pill from US$7.35 to US$0.19 to US$0.22 per pill, which represents a 30-fold difference in price.^6^ Within 5 years of offering letrozole, docetaxel, imatinib, and erlotinib in the public health system, an additional 8,916 patients received letrozole, 10,813 were treated with docetaxel, 1,846 with imatinib, and 256 with erlotinib.^6^

The first compulsory license for an oncology drug in India was issued in 2012 for sorafenib. At that time, Bayer’s sorafenib was used primarily for advanced liver and renal cancer and improved outcomes only by a few months; however, a year of treatment cost US$96,000.^6^ By pursuing a compulsory license, generic manufacturing of sorafenib was started in India, which reduced the cost of treatment to US$2,124 for 1 year—US$177 per month from US$8,000 per month.^6^

Since 2013, the government of India has pursued compulsory licenses for trastuzumab, dasatinib, and ixabepilone. As a result, Roche abandoned its patent claims for trastuzumab, and the Indian high court approved a local drug company, Biocon, to produce a biosimilar^34^; however, Roche subsequently sued the Indian drug regulatory agency for approving Biocon-Mylan’s trastuzumab as a biosimilar without carrying out clinical trials.^35^ Currently, Biocon has entered phase III trials with trastuzumab to demonstrate that their biosimilar version of trastuzumab has efficacy against human epidermal growth factor receptor 2 (HER2) –positive breast cancer.^36^ In our opinion, trastuzumab is a good choice for a compulsory license as it has excellent efficacy against HER2-positive breast cancer in both the metastatic and early disease settings. However, as the case from India shows, the process for obtaining a compulsory license and identifying a manufacturer to support the drug’s development can take years and delay access to important medications.

For dasatinib, the Indian patent office rejected the request for a compulsory license, saying that the government failed to explore the proper channels to obtain a voluntary license from the patent holder.^37,38^ A request for a subsequent compulsory license was pursued in 2015 and the Delhi High Court rejected the request and upheld the patent held by Bristol-Myers Squibb.^37,38^ The compulsory license request for ixabepilone was withdrawn as a result of toxicity concerns related to the drug.^39^

From an economic perspective, substitution of patented drugs with generic versions is cost saving, and from a public health standpoint, not only permitting but also encouraging generic drug production and use increases access to essential cancer medications in LMICs. For example, in India, if generic versions of paclitaxel, docetaxel, gemcitabine, oxaliplatin, and irinotecan—five commonly used chemotherapeutic agents—were introduced, the potential annual savings for the health care system is nearly US$843 million (or €670 million).^40^ In fact, generic versions of these drugs are already available and cost 8.9% to 36% less than the equivalent branded drug, and there is only a need to permit their use in the Indian market.^40^

## IS THERE A ROLE FOR COMPULSORY LICENSES TO IMPROVE ACCESS TO CANCER MEDICATIONS?

Although some critics have suggested that the failure to uphold intellectual property rights will decrease incentives for innovation and, therefore, lead to fewer new medications in the future, evidence in support of this notion is scant. In fact, > 80% of financial gains from cancer drugs comes from high-income countries in which compulsory licensing is rarely used or approved.^13^ One observational study investigated this issue and found that pharmaceutical companies affected by compulsory licenses did not have a decline in the rate of new medicines patented or their measured inventive and innovation activity.^41^

Obviously, some pharmaceutical companies view compulsory licenses as a threat to their intellectual property, research and development, and medication sales. There have been a few cases in which the pharmaceutical industry has tried to pressure countries to deter them from issuing compulsory licenses. For example, Pfizer announced that it would rethink investments in Egypt after the country issued a compulsory license for sildenafil in 2002^42^; however, this is unusual, and pharmaceutical investment and growth continues in many countries, such as Brazil and South Africa, where compulsory licenses have been issued.^13^

In place of using compulsory licensing, LMICs may benefit from negotiating directly with the patent owner, that is, pharmaceutical companies, to offer essential drugs to their populations. In many cases, pharmaceutical companies have offered affordable prices for medications, if ordered in bulk, to serve a large population, which is an approach that we support. However, there are no data on how long these negotiations take before a decision is made and if pharmaceutical companies purposefully delay this process so that an agreement or a compulsory license is not immediately granted.

There is also the argument that the price of patented medications is not the main barrier to medication access in LMICs and, in fact, that lack of manufacturing capacity or poor health care systems are larger contributors that impact access.^10,15^ Counter arguments to this point are simple: if LMICs save money on medication expense, then these savings can be invested in improvement of their own drug manufacturing capacity and health systems. In Thailand, a study found that if relevant HIV/AIDs drugs were not patented, an additional 10,000 prescriptions could be made, which would increase access by 50%.^10^

The high costs of cancer drugs threaten access to cancer treatment even in high-income countries. As a result of its extremely high cost, trastuzumab emtanzine (T-DM1), a drug used to treat metastatic HER2-positive breast cancer, has not been made available to patients treated in the national health system in the United Kingdom, according to a recent recommendation by the National Institute for Health and Care Excellence. The National Institute for Health and Care Excellence estimates that only 1,500 women in the United Kingdom would benefit from treatment with T-DM1 every year and that a year of treatment costs £102,405, roughly 3.9 times the 2014 per capita income of £26,350 in the United Kingdom.^43,44^ Compared with lapatinib plus capecitabine therapy in this setting, T-DM1 costs £166,400 per quality-adjusted life year (QALY) gained,^45^ which is significantly higher than the cost-effectiveness threshold in the United Kingdom of £30,000/QALY gained.^46^ In contrast to lapatinib and capecitabine, T-DM1 therapy has a more favorable adverse effect profile and is generally well tolerated, an important consideration in patients with advanced cancer where preserving quality of life is a major goal; this fact is not accounted for in the cost and QALY calculation.

Out of concern of the access barrier to T-DM1 therapy, the Coalition for Affordable T-DM1, a civil organization, sent a formal letter to United Kingdom secretary of state for health to ask that the government use provisions in United Kingdom patent laws to authorize the manufacture or importation of generic versions of T-DM1 without the permission of Roche.^43,44^ This case simply exemplifies the exorbitantly high price of cancer medications and the urgent need to find solutions to this problem, especially in resource-conscious or resource-constrained settings.

## BARRIERS TO COMPULSORY LICENSES

Pursuing a compulsory license does seem to be a possible solution to improve access to medications in LMICs. Nonetheless, there are significant barriers to this route for many LMICs and, consequently, this route has not been frequently pursued for cancer drugs since the introduction of the TRIPS Agreement and Doha Declaration in 2001 for essential cancer medications. A 2005 report by the WHO found that many LMICs did not implement many TRIPS flexibilities into their legislation—compulsory licensing, parallel importation, limits on data protection, use of broad research, and other exceptions to patentability—and this was primarily attributed to a lack of legal and technical expertise needed to draft such legistlation.^47^ Furthermore, even when compulsory licenses are approved, there can still be delays in the introduction of these generic medications into the market. For example, in Thailand, introduction of generic medications was delayed for 1 year from the time compulsory licenses were approved.^32^ The reason for this was that some patent-holding companies alleged that importation and production of generic drugs under Thailand’s government-use licensing policy, a form of compulsory licensing, was not protected by TRIPS/DOHA. As a result, this caused confusion and reluctance among generic producers and, consequently, a delay in importing the medications.^32^ In recognition of these issues, efforts are needed to resolve these barriers in obtaining compulsory licenses for essential medications.

## ALTERNATIVE STRATEGIES TO IMPROVE ACCESS TO CANCER MEDICATIONS

New strategies are being considered to ensure that cheaper medicines flow to countries most in need. These include tiered price schemes, public–private partnerships, patent pools, and tax incentives.^48,49^

Tiered pricing, also known as price discrimination and differential or equity pricing, consists of charging different prices for the same product or service in different markets or segments of a market. The method usually is based on consumer ability to pay and not necessarily on the market demand. For example, medications will be more expensive in high-income countries and more affordable in LMICs.

In the last two decades, tiered pricing has been frequently used to lower the cost of vaccines and AIDS medications in LMICs. Until recently, though the situation has started to change, tiered pricing had not been applied frequently to oncology medications. For example, in 2011, there was little variation in price between widely used cancer medications—oxaliplatin, bevacizumab, cetuzimab, trastuzumab, sorafenib, erlotinib, and gefitinib—in Southeast Asia despite gross national income per capita varying between countries by three-fold or more.^5^ A few pharmaceutical companies have introduced tiered pricing for their cancer products in the last few years, including GlaxoSmithKline, Eli Lilly, Sanofi, and Roche.^5^

One effort underway to promote tiered pricing has been organized by the Global Fund and GAVI Alliance. This effort is known as the Equitable Access Initiative and aims to improve global access to essential medicines.^50^ Stratifying prices into tiers can be done in several ways. The group’s first proposal recommended prices be set by an international public health body, such as the WHO; however, a more recent proposal considers voluntary discounts by pharmaceutical companies, and this could lower prices even further.^50^

Despite these efforts, it is unclear whether stratifying prices would significantly reduce the cost of cancer drugs. A study from the President’s Emergency Plan for AIDS Relief in 2005 concluded that tiered pricing had failed to lower the price of on-patent HIV/AIDS medications, and that generic formulas were primarily responsible for reducing the price of AIDS drugs and improving access.^50^ From 2004 to 2008, the average price of HIV antiretrovirals decreased by 48%, and it is debated whether this is primarily a result of tiered pricing, generic manufacturing, targeted negotiations, or a combination of the three.^7^ On one hand, because of these controversies, the Equitable Access Initiative and other organizations, such as Médecins Sans Frontières, do not endorse tiered pricing as they worry that this may result in middle-income countries paying higher prices for medicines.^25,50,51^ On the other hand, some believe tiered pricing could work well in small-volume markets or in regions with uncertain production capacities on which an occasional short-term solution is needed to temporarily secure access to a medication.^51^

Whatever solution is pursued, there is growing recognition that the price of oncology medications is a problem. A recent joint symposium that involved the WHO, WTO, and the World Intellectual Property Organization agreed that prices for essential medicines continue to be an issue in LMICs, and concerns over how to increase access to medicines were discussed.^30^ In 2015, the WHO updated their list of essential medicines and included 16 new oncology drugs, several of which are under patent and are priced high (Table 2).^52-54^

**Table 2:**
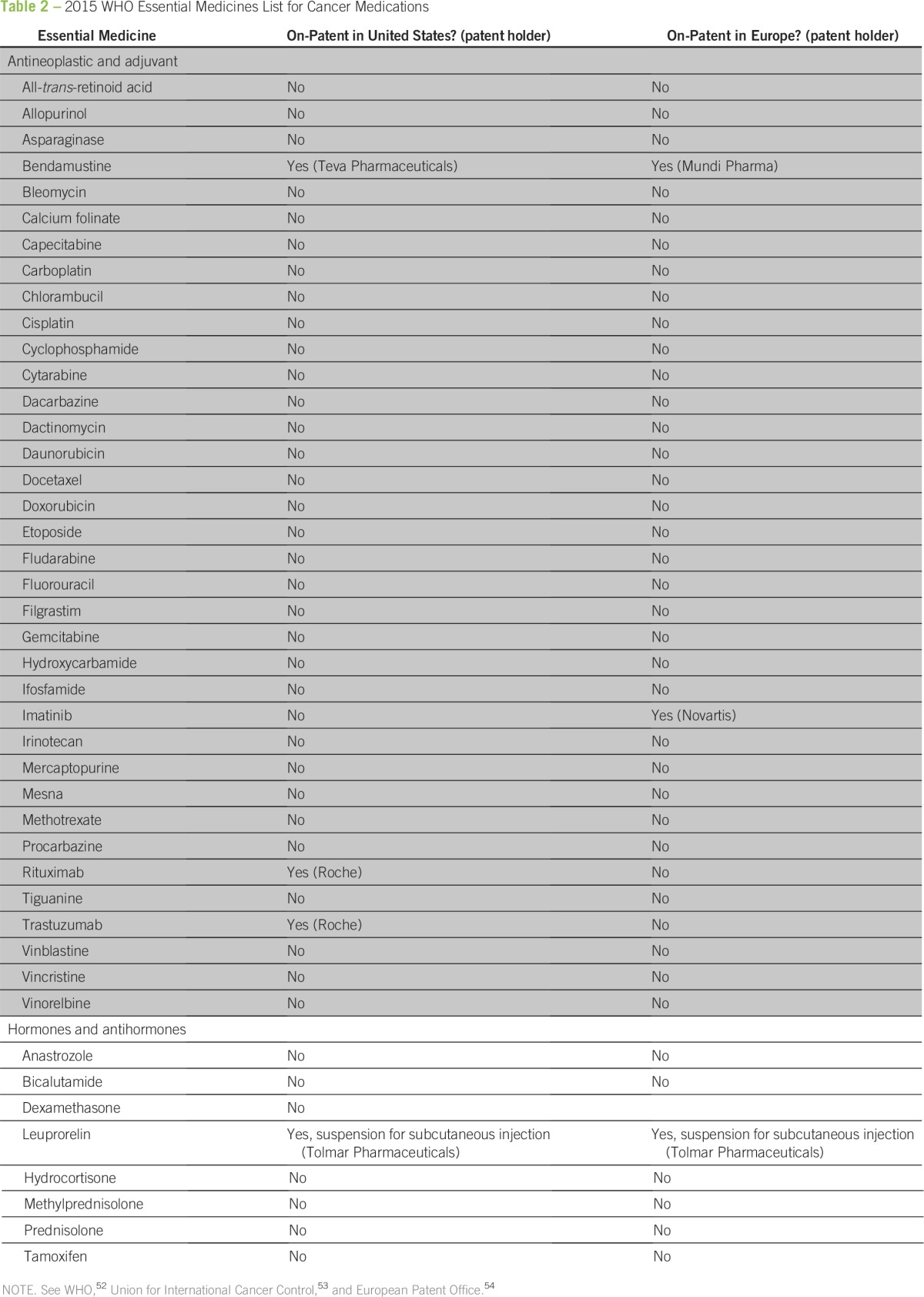
2015 WHO Essential Medicines List for Cancer Medications

In conclusion, price negotiations with pharmaceutical companies and use of compulsory licenses, albeit controversial, has led to an important advance in access to drugs for communicable diseases, such as HIV and hepatitis C and, more recently, for cancer, in LMICs. Even the threat of compulsory licenses can work as a bargaining chip when discussing drug prices with industry. Indeed, the pharmaceutical industry has responded with a series of price-discrimination and market-access strategies to increase sales and access to medications in LMICs, as seen in HIV treatment access in the last decade. In this context, it is imperative that we review existing approaches to drug pricing and identify solutions to improve access to cancer medications in emerging markets and LMICs. We will have the most success solving this problem through collaboration between the pharmaceutical industry, governments, private funds, civil organizations, and health professionals; only then will we be able to best control the growing burden of cancer worldwide.^55^
